# Intelligence

**Published:** 1829-07

**Authors:** 


### INTELLIGENCE.

---

### MONTHLY REPORT OF PREVALENT DISEASES.

As far as we can judge from our own opportunities of observation, both in hospital and private practice, no particular disease has occurred so frequently since our last report as to justify the application of the term "prevalent."

The following case came under our notice a few days ago. We were desired to visit, without delay, a little girl, about four years old, who was said to be dying. We found her in the following state: Countenance very pallid; convulsive twitchings of the muscles of the face; skin cold; respiration short and laborious; occasional attempts to vomit; pulse nearly imperceptible. Up to the moment of the occurrence of these symptoms, the child had been in perfect health. The cause of so sudden an attack was, of course, obscure. The friends were not aware that any thing had been eaten which was likely to disagree with the stomach. A small quantity of warm white wine and water was given immediately, and the child rallied a little. In about half an hour she vomited freely, and threw up, together with the ordinary food she had taken for dinner, a large quantity of the flowers of the common liburnum, which she had eaten unknown to her attendant. For several hours she remained very languid, and on the next day appeared to be perfectly recovered.

---

*Infringement of the Act of Parliament for Regulating the Practice of Apothecaries.*

*Apothecaries' Hall; May* 1829.

Whereas the Master and Wardens of the Society of Apothecaries have received complaints that many persons are practising as apothecaries, both in the metropolis and other parts of England and Wales, contrary to the Act of Parliament for better Regulating the Practice of Apothecaries; but the information thus received having been frequently defective and inaccurate, they have not been able, in all cases, to take the necessary steps to enforce observance of the Act of Parliament; they therefore earnestly request that all persons having knowledge of any infringement of the said Act, by unqualified persons practising as apothecaries in any part of England and Wales, will furnish them with such full particulars and precise information as will enable them to take the necessary steps to protect regular practitioners, as well as the public, from the mischief arising from unqualified or incompetent persons acting as apothecaries, and to enforce the law by bringing the offenders to justice.

It is requested that all such information may be communicated either by letter addressed to the Clerk of the Society, Apothecaries' Hall, London, or to the Clerk personally, at his office at the Hall, on Tuesdays and Thursdays, between the hours of one and three o'clock; where printed particulars of the requisite points of information may be obtained.

The Master and Wardens think it right to give this public caution to parents and guardians, before they article their sons or wards to apothecaries or surgeon-apothecaries, to make strict inquiry whether such apothecaries or surgeon-apothecaries are themselves qualified to practise according to the provisions of the said Act of Parliament; as no indentures of apprenticeship to persons *not* so qualified can be received by the Court of Examiners of the Society.

Information on this subject may be obtained by addressing a letter to the Clerk of the Society, or by personal inquiry as above mentioned.

The attention of the public is particularly requested to the 21st section of the Act, which enacts that an apothecary cannot recover any charges claimed by him, unless qualified to act as such under the provisions of the said Act.

(By order,) Edmund Bacot, *Clerk.*

N.B. Anonymous communications of any kind cannot be attended to.

---

*Apothecaries' Hall.*—Candidates who present themselves for examination, and whose studies either commenced or were completed prior to the 1st of February, 1828, will be required to produce the following testimonials of their attendance:

1. One course of lectures on Chemistry;
2. One course of lectures on Materia Medica;
3. Two courses of lectures on Anatomy and Physiology;
4. Two courses of lectures on the Theory and Practice of Medicine;
5. Six months' physician's practice at an hospital, or nine months at a dispensary.

The above curriculum is the same as that originally enjoined in 1815, with the addition of three months' dispensary practice.

---

Dr. Dewees, of Philadelphia, is preparing for publication a System of Practical Medicine. We understand it will be strictly a practical book, divested as far as possible of theoretical disquisitions, and will contain the result of nearly forty years' experience. The industry and talent evinced by Dr. Dewees in all his former publications, lead us to look forward to this promised work with the expectation of finding in it much valuable matter, especially to English physicians, who must feel anxious to instruct themselves in the pathological and practical views of their trans-atlantic brethren.

*St. George's Hospital.*—Dr. WILSON has been unanimously appointed physician to St. George's Hospital, in the room of Dr. YOUNG.

### MEETINGS OF THE COLLEGE OF PHYSICIANS.

### MAY 18TH.

*First Paper: on some extraordinary Cases of Metastasis of Asthma into Derangement.* By Dr. TURNER.

The first case was that of a gentleman, who, at the age of fifty, began to suffer frequent attacks of asthma, which continued becoming worse till seventy, when his breathing became suddenly easy, every symptom of dyspnœa vanishing; but he was seized with painful priapism, and decided aberration of mind. These symptoms, however, observed, to a certain degree, the form and habitude of his asthma, appearing in paroxysms of from six to eight hours' duration.

This state lasted for upwards of a month, when he gradually sunk, and, for the last twelve hours of his life, he became quite rational.

The next was the case of a lady, in whom, towards the close of her life, the asthmatic symptoms appeared to yield to an attack of mental delusion.

Dr. TURNER then adverted to a case of similar metastasis related by Dr. WITHERING, in his Treatise on the Use of the Foxglove; and stated also that the president of the College, Sir HENRY HALFORD, had communicated to him that, during the epidemic influenza of 1802, he had attended a lady, with Dr. BAILLIE, who had been subject to attacks of asthma for many years, and who suffered so much at that time as to be for several days in the utmost danger. Sir Henry, happening himself to be confined for a short time to his own house, was surprised, on going out again and inquiring after this patient, to find that she was labouring under derangement, but relieved of all her difficulty of breathing. The derangement continued six weeks, after which the asthmatic symptoms returned; and she lived four years afterwards, quite free from any mental delusion.

Dr. Turner thought that these cases strongly confirmed an opinion advanced by Dr. HEBERDEN, in his Commentaries, that asthma must be considered "to be, in every instance, occasioned by a disturbance of those functions which are attributed to the nerves." He adverted particularly to the condition of sleep, which brings on a fit of asthma, and is also known to be one of the most common exciting causes of other nervous disorders, as epilepsy, paralysis, &c.

*Second Paper: Suggestions for preventing the Spreading of Contagion in Gibraltar.* By Mr. JEFFERY, the Engineer, whose plans for making approaches to the new London Bridge have been adopted by Parliament.

Sir HENRY HALFORD informed the meeting that he had hoped to have been able, by this time, to have laid before them the Report of the Board appointed to inquire into the late epidemic fever in that garrison, but it had not yet reached England.

The suggestions of Mr. JEFFERY, who had been long resident in Gibraltar, consisted chiefly of plans for elevating a sufficient quantity of sea water, by means of steam-engines, to cleanse out the sewers and wash the streets; and also of hints for cooling and ventilating the parched side of the rock upon which the town is built, by perforations through the rock from east to west.

JUNE 1ST.

*Case of Tic Douloureux, by the celebrated* JOHN LOCKE.

A literary curiosity of great interest was laid before the meeting: a case detailed by the celebrated JOHN LOCKE. This curious document was obtained by Dr. C. M. CLARKE, from Lord KING, and presented to the College. The original manuscript was laid upon the table, and consisted of a French Almanack, bound up with a number of leaves which had been originally blank, but which were filled with various notes and memoranda in the handwriting of Locke, and among others the case in question.

It has often been doubted whether Locke ever practised as a physician, but the question is now set at rest. In Lord GRENVILLE'S pamphlet, entitled "Oxford and Locke," he remarks that "in the printed life of Locke, commonly prefixed to his works, we are told that he applied himself, at the University, with great diligence to the study of medicine, 'not with any design of practising as a physician, but principally for the benefit of his own constitution, which was weak." His lordship goes on to observe, that no such motive is ascribed to Locke by Le Clerc, from whom our knowledge of his private history is principally derived; nor, indeed, is the supposition at all probable. Le Clerc, however, asserts "that Locke never practised physic for profit, though he was highly esteemed by the ablest physicians of his time." In proof of this, we need only quote the following passage from SYDENHAM: "Nosti preterea quam huic meæ methodo suffragantem habeam, qui eam intimius per omnia perspexerat utrique nostrum conjunctissimum, Dominum Joannem Locke; quo quidem viro, sive ingenio judicioque acri et subacto, sive etiam antiquis, hoc est, optimis moribus, vix superiorem quemquam, inter eos qui nunc sunt homines, repertum iri confido, paucissimos certè pares."

Lord Grenville says, that the assertion that Locke had never actually practised is "unquestionably erroneous;" and the case which we subjoin proves the correctness of his opinion.

Locke was called to see the Countess of Northumberland, who was the ambassadress at Paris, December 2, 1677. The case was evidently one of tic douloureux. It is entitled *Convulsio*, and the symptoms are thus described: Acute pain over the right cheek up to her ear; in the intervals, pain in the teeth. She was warned of the approach of the fits by a throbbing she felt in the lower jaw, where she had had a tooth drawn the previous summer. The fits had been preceded by three or four days of ordinary toothach. There was no swelling or inflammation, no flux of rheum, no external swelling, no indication for bleeding; besides which, that remedy had been tried some months before, without effect.

"It being night," says Locke, "I thought at present there was nothing to be done but to give her ladyship present ease by some topical application." He thought first of a blister, but paused till he had made some more general evacuation. He therefore ordered an opiate embrocation to the gums, which gave her much relief. On the following day (for the case is related in the form of a journal,) he again deliberated about the propriety of the exhibition of an aperient, but the extreme cold weather made him conclude in the following manner: "I apprehended that a purge, which I thought very necessary, would be dangerous in such a season, because, if weak, it might cause disorder, with very little or no evacuation; if strong, in so delicate a constitution I could not tell how to venture; beside that, I feared she might take cold in the working, which might increase the mischief."

The result of his prudent caution was, that he prescribed a drop of Æthereum Terebinthinæ on a little lint, which she applied to the gap whence the tooth had been extracted, but it did not allay the pain; and he then ventured upon the purge, and gave a mercurial one, which "wrought very well seven or eight times."

After the operation of this medicine he prescribed an opiate draught, and during the following night she enjoyed some sleep. With occasional exacerbations, the fits upon the whole began gradually to abate in severity. He describes most accurately what we all know to be the truth in this cruel disease, how various slight causes bring on the paroxysm of pain; how touching any part of the affected side of the body (even the foot of that side), talking, or opening her mouth to eat, brought on the twitches of pain. He reasons upon this strange nervous affection very sensibly, considers what the original mischief was, and how far the extraction of the tooth had to do with the increase of the malady; and concludes that the root of the mischief lies in some harm done to the nerve connected with the tooth. The tooth itself, when it was drawn, was found to be a sound one, and its extraction so far from a remedy that it increased the violence and frequency of the fits. Locke continued in attendance till December 16th, a space of a fortnight, when he pronounced the lady ambassadress "quite well."

On Monday, Dec. 20th, he writes in his MS.:

"Memorandum: that my lady ambassadrice's gums itched vehemently after the pain was gone, and did so for several days after; and used to do so for several years before any tooth was drawn."

*Observations on Insanity.* By Sir H. HALFORD.

After the above had been read,

Sir HENRY HALFORD stated, that, in consequence of having understood that there was no paper for the present evening, (for Locke's case had only just been received,) he had hastily thrown together some observations on insanity. As there was sufficient time left, he would read them to the meeting.

Sir Henry observed, that, in the closet scene in "Hamlet," the following words occur:

"——Ecstasy!
My pulse, as yours, doth temperately keep time,
And makes as healthful music; 'tis not madness
That I have uttered; bring me to the test,
And I the matter will reword, which madness
Would gambol from."

The circumstance to which the learned President particularly alluded, was the expression "I the matter will *reword*;" and he proceeded to relate the following case, in illustration of the justness of SHAKSPEARE's "test." He was called, last January, to a gentleman then in a state of mental derangement. A short time previous to his illness, he had sent for his solicitor, and given directions about his will. He stated his intention of adding 500*l.* a year to his mother's jointure, and of leaving various legacies; adding that his friend, the solicitor, was to be the residuary legatee. The solicitor, in the most honourable manner, told him that he could not consent to the last part of the arrangement, unless at the end of six months he continued of the same mind upon the subject. In the interval, he was attacked with mental excitement, for which he was attended by Sir Henry Halford and Sir G. Tuthill. One day, on asking him how he did, he appeared calm and collected, and answered that he was very ill, and only anxious to settle his affairs and make his will. Next day he repeated the same expressions, in a tone and manner which induced his attendants to comply with his request, and the solicitor was sent for, who brought with him a will drawn up according to the instructions he had formerly received. This was read over to the gentleman; and being asked, after each clause, if such was his meaning, he distinctly replied "Yes, yes." The will was then executed, being witnessed by his physicians. On going down stairs, Sir Henry observed upon the unpleasant circumstance of the medical attendants becoming involved in a deed which was likely to become the subject of litigation, and proposed that they should return to him, and apply Hamlet's test, by ascertaining whether he could "reword" his will. With regard to several of the clauses, this was the case; but he stated that he had left one individual 10,000*l.*, whereas he had only left him 5000*l.*; and, on being asked to whom the residue of his fortune was to go, he answered "To the heir at law, to be sure!" Being asked who was the heir at law, he replied that he did not know. Thus, said Sir Henry, he could not "reword" his meaning, but "gamboled" from the matter.

The author then adverted to the fidelity of the pictures drawn by Shakspeare, so justly characterized by Johnson as the poet of nature. He also alluded to the writings of the ancient poets, as containing many descriptions which might be recognised by an attentive observer. He had himself seen two of the cases mentioned by Horace illustrated to the very life. One, a man of high rank, supposed himself present at a theatrical entertainment, and Sir Henry had heard him urging Garrick to exert himself in the part of Hamlet, which he supposed him then to be acting. The other case was that of a gentleman of large fortune who possessed himself of every thing he could get, but parted with nothing. He was brought from the court of King's Bench, having refused to pay for a picture which he had bought, and which was valued at 1500*l.* Sir Henry told the jury, that if they would go to the gentleman's house in Portland place, they would find 50,000*l.* worth of property: amongst the rest this very picture, with baby-houses and baubles strewed over his dining-room.

The paper was listened to with great interest, and this was increased by the very animated manner in which it was read by the learned president.

---

### MONTHLY LIST OF MEDICAL BOOKS.

### METEOROLOGICAL JOURNAL,

By Messrs. HARRIS and Co. Mathematical Instrument Makers, 50, High Holborn.

| May | Rain gauge. | Moon. | Thermom. 9A.M. | Thermom. MAX. | Thermom. MIN. | Barometer. 9 A.M. | Barometer. 10P.M. | De Luc's Hygrom. 9A.M. | De Luc's Hygrom. 10P.M. | Winds. 9 A.M. | Winds. 10P.M. | Atmospheric Variations. 9 a.m. | Atmospheric Variations. 2 p.m. | Atmospheric Variations. 10 p.m. |
|---|---|---|---|---|---|---|---|---|---|---|---|---|---|---|
| 20 | | | 63 | 69 | 52 | 29.85 | 29.90 | 44 | 44 | ENE | ENE | Fine | Fine | Fine |
| 21 | | | 65 | 69 | 46 | 30.00 | 30.02 | 44 | 45 | ENE | NE | — | — | — |
| 22 | | | 58 | 68 | 50 | .05 | .06 | 46 | 47 | NE | E | — | — | — |
| 23 | | | 60 | 72 | 52 | .10 | .10 | 47 | 44 | E | SSE | — | — | — |
| 24 | | | 64 | 75 | 47 | .17 | .22 | 45 | 45 | ENE | NW | — | Rain | Cloudy |
| 25 | .14 | ☾ | 52 | 63 | 44 | .29 | .30 | 45 | 50 | NE | NNE | — | Fine | Fine |
| 26 | | | 60 | 63 | 47 | .28 | .22 | 50 | 50 | ENE | NNE | — | — | — |
| 27 | | | 61 | 67 | 50 | .17 | .16 | 50 | 45 | NE | NE | — | — | — |
| 28 | | | 62 | 69 | 50 | .07 | .07 | 40 | 45 | NE | NE | — | — | — |
| 29 | | | 60 | 67 | 50 | .08 | .08 | 47 | 46 | NE | NE | Cloudy | Fine | Fine |
| 30 | | | 56 | 65 | 49 | .08 | .06 | 45 | 46 | NE | NE | Overca. | Cloudy | Cloudy |
| 31 | | | 57 | 65 | 48 | .09 | .12 | 48 | 48 | SW | W | Fine | Fine | Cloudy |
| June | | | | | | | | | | | | | | |
| 1 | | ● | 56 | 69 | 52 | .13 | .16 | 49 | 47 | NNE | N | Cloudy | Fine | Fine |
| 2 | | | 65 | 72 | 61 | .19 | .15 | 46 | 46 | NNW | NNW | Fine | Fine | Fine |
| 3 | | | 69 | 77 | 62 | .12 | .02 | 46 | 43 | NNW | NNW | — | — | — |
| 4 | | | 68 | 73 | 57 | .02 | 29.87 | 43 | 40 | NNW | NNW | — | — | — |
| 5 | | | 65 | 68 | 47 | 29.91 | 30.01 | 40 | 40 | NNW | NNW | — | — | — |
| 6 | | | 57 | 65 | 45 | 30.13 | .15 | 40 | 42 | N | NE | — | — | — |
| 7 | | | 54 | 63 | 43 | .19 | .23 | 42 | 44 | NW | NW | Cloudy | Fine | Fine |
| 8 | | | 50 | 59 | 47 | .23 | .24 | 44 | 44 | NNE | NE | Show'ry | Cloudy | Fine |
| 9 | .09 | ☽ | 57 | 64 | 47 | .23 | .22 | 45 | 43 | NNE | NE | Cloudy | Fine | Fine |
| 10 | | | 58 | 66 | 48 | .27 | .27 | 43 | 45 | NE | ENE | Cloudy | Fine | — |
| 11 | | | 57 | 67 | 52 | .00 | .23 | 45 | 49 | ENE | ENE | Fine | — | — |
| 12 | | | 60 | 72 | 55 | .23 | .20 | 49 | 45 | ENE | ESE | — | — | — |
| 13 | | | 68 | 73 | 55 | .19 | .14 | 45 | 45 | SE | S | — | — | — |
| 14 | | | 67 | 75 | 57 | .08 | .03 | 45 | 44 | SW | SW | — | — | — |
| 15 | | | 68 | 71 | 67 | .00 | 29.84 | 44 | 42 | SW | SW | — | — | — |
| 16 | | | 65 | 67 | 50 | 29.72 | .74 | 42 | 43 | W | WSW | Cloudy | Show'ry | Fine |
| 17 | .10 | ○ | 57 | 64 | 52 | .75 | .75 | 43 | 43 | WSW | WNW | Cloudy | Fine | Cloudy |
| 18 | | | 61 | 68 | 53 | .79 | .91 | 43 | 44 | NW | NE | Cloudy | Cloudy | Cloudy |
| 19 | | | 65 | 74 | 56 | .92 | .87 | 44 | 40 | SE | SSW | Fine | Fine | Fine |

The quantity of Rain fallen in the month of May, was 14-100ths of an inch.

